# Clinical Implementation and Evaluation of Auto-Segmentation Tools for Multi-Site Contouring in Radiotherapy

**DOI:** 10.1016/j.phro.2023.100515

**Published:** 2023-11-17

**Authors:** Gerd Heilemann, Martin Buschmann, Wolfgang Lechner, Vincent Dick, Franziska Eckert, Martin Heilmann, Harald Herrmann, Matthias Moll, Johannes Knoth, Stefan Konrad, Inga-Malin Simek, Christopher Thiele, Alexandru Zaharie, Dietmar Georg, Joachim Widder, Petra Trnkova

**Affiliations:** Department of Radiation Oncology, Comprehensive Cancer Center Vienna, Medical University Vienna, Vienna, Austria

**Keywords:** Auto-segmentation, Segmentation, Deep Learning, Radiotherapy

## Abstract

**Background and purpose:**

Tools for auto-segmentation in radiotherapy are widely available, but guidelines for clinical implementation are missing. The goal was to develop a workflow for performance evaluation of three commercial auto-segmentation tools to select one candidate for clinical implementation.

**Materials and Methods:**

One hundred patients with six treatment sites (brain, head-and-neck, thorax, abdomen, and pelvis) were included. Three sets of AI-based contours for organs-at-risk (OAR) generated by three software tools and manually drawn expert contours were blindly rated for contouring accuracy. The dice similarity coefficient (DSC), the Hausdorff distance, and a dose/volume evaluation based on the recalculation of the original treatment plan were assessed. Statistically significant differences were tested using the Kruskal-Wallis test and the post-hoc Dunn Test with Bonferroni correction.

**Results:**

The mean DSC scores compared to expert contours for all OARs combined were 0.80 ± 0.10, 0.75 ± 0.10, and 0.74 ± 0.11 for the three software tools. Physicians' rating identified equivalent or superior performance of some AI-based contours in head (eye, lens, optic nerve, brain, chiasm), thorax (e.g., heart and lungs), and pelvis and abdomen (e.g., kidney, femoral head) compared to manual contours. For some OARs, the AI models provided results requiring only minor corrections. Bowel-bag and stomach were not fit for direct use. During the interdisciplinary discussion, the physicians' rating was considered the most relevant.

**Conclusion:**

A comprehensive method for evaluation and clinical implementation of commercially available auto-segmentation software was developed. The in-depth analysis yielded clear instructions for clinical use within the radiotherapy department.

## 1 Introduction

The contouring of organs-at-risk (OARs) and target volumes on computed tomography (CT) and magnetic resonance (MR) images is an essential task in radiotherapy. It is very resource-intensive, particularly when done manually. Moreover, the results of manual contouring are subject to inter- and intra-observer variation [1] and are affected by the user’s level of experience [2]. These variations may significantly impact a dose/volume-based plan evaluation and clinical outcome [3] or lead to a bias in clinical trials [4].

Auto-segmentation was designed to address these shortcomings of manual contours by providing much faster and user-independent results. Large-scale clinical implementation offers the potential to improve standardization across institutes and users [4]. Recently, artificial intelligence (AI) models have complemented or superseded conventional auto-segmentation methods such as atlas-based models [5], [6], enabling fast progress in the field [7], [8], [9], [10]. Many vendors offer pre-trained models that can be readily utilized in clinics. Not only does this present a significant opportunity for standardization, but it also paves the way toward online adaptive radiotherapy.

AI-based auto-segmentation models are usually a “black box”. This poses a challenge for clinics when integrating AI tools into radiation oncology, because the model interpretability is very difficult and the results dependent on the data used for the training [11]. Moreover, commercial models are usually based on data from other institutes. Therefore, performing an extensive evaluation of auto-segmentation prior to clinical implementation to understand their accuracy and limitations is crucial. Previous studies have focused on single sites [6], [10], [12], [13], [14], [15], [16], [17]. This study aimed to develop a comprehensive procedure for evaluating AI-based auto-segmentation software for all sites before clinical implementation.

## 2 Methods

### 2.1 Patients and inclusion criteria

This study was approved by the Institutional Review Board of the Medical University of Vienna (EK 1733/2022). Six different treatment sites were selected to cover most patients undergoing radiation therapy at our department: brain, head and neck, thorax, abdomen, male pelvis, and female pelvis. A representative subset of 15–20 patients per site was retrospectively selected and pseudonymized with a total of 100 patients. The selection parameters are listed in Table 1. Each patient data set contained planning CT, clinical contours, and clinical treatment plans. To reflect the real clinical practice, no improvement of contours was performed prior to the analysis.

**Table 1:** An overview of patient cohorts for each treatment site. Each patient was rated by different MDs, indicated by initials. Additional manually delineated contours were added by the MDs indicated in the column under ‘Extra contours’. * For the head and neck site only 12 contours were rated by MDs, but all 15 were analyzed geometrically and with respect to dose/volume metrics.

Site	Types of cancer	Number of patients	Rated by # MDs	Extra contours (by # MDs)	Slice thickness (mm)
Brain	gliomas, metastases, eye	15	2 (FE, CT)	–	2
Head and neck	Nasopharynx, oropharynx, tongue, larynx	15*	2 (HH, IMS)	–	4
Thorax	Breast, lung, ribs	15	2 (SK, MH)	–	2 and 4 (lung)2 (breast)
Abdomen	Pancreatic, liver, digestive tract	15	2 (AZ, VD)	15 (AZ, VD)	4
Pelvis (female)	cervix, vagina, endometrium, bladder, ano-rectal	20	3 (JK, IMS, MM)	10 (IMS, JK, MM)	4
Pelvis (male)	Primary & post-operative prostate, bladder, ano-rectal	20	3 (AZ, JK, MM)	10 (IMS, JK, MM)	2 (prostate) and 4 (others)

The CT scans were performed with the standard site-specific clinical CT protocols (Siemens Somatom Definition AS) with a tube voltage of up to 120 kVp, slice thicknesses 2 to 4 mm and in-plane resolution of approximately 1 mm.

## 3 Reference contours and auto-contours

The clinical contours were used for all treatment sites to benchmark the contours produced by the auto-segmentation models. Most contours were manually delineated on CT by radiation technologists (RTTs) and checked by the radiation oncologist. In male pelvis, the physician delineated all OARs, and in the brain, some contours were delineated on rigidly registered MR images.

Three different commercially available software tools were evaluated. They will be referred to as Software A, B, and C throughout this study (see Supplementary Data I).

### 3.1 Workflow of evaluation of auto-segmentation software and clinical implementation

**Fig. 1:**
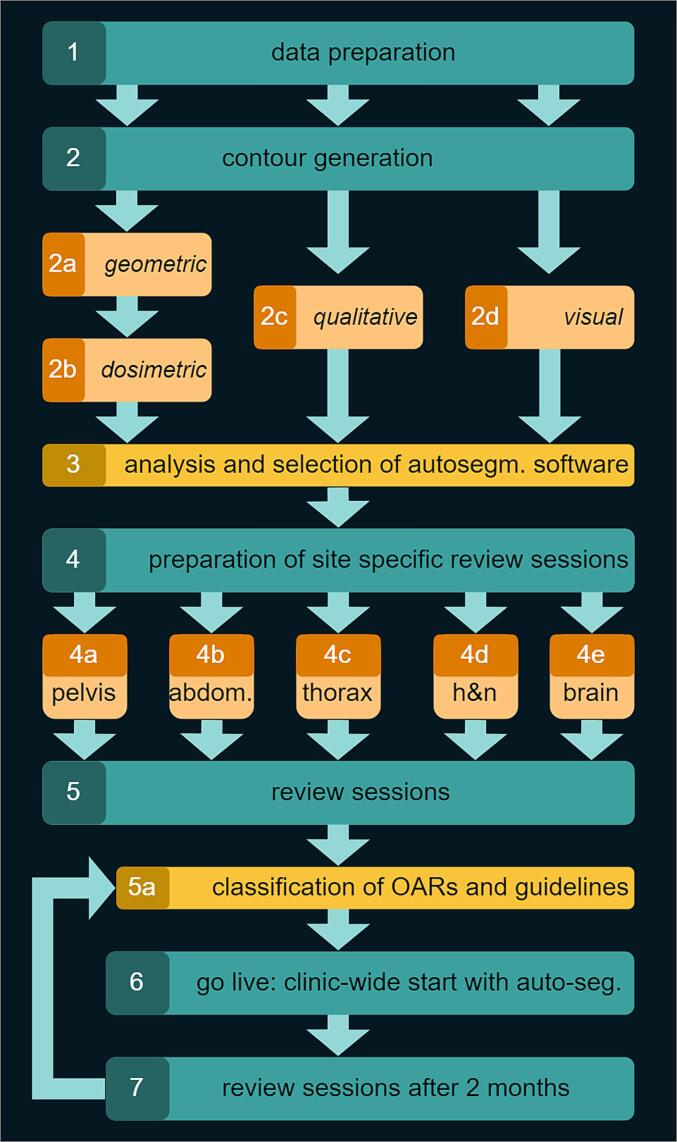
Flowchart of the implementation and selection of auto-segmentation software in clinical practice.

Evaluating software candidates for clinical auto-segmentation was a comprehensive and collaborative effort in multiple phases over several months (Fig. 1). A team of medical physicists handled the preparation and execution of the whole process.

In the first phase, quantitative geometric, dose/volume metric-based, and qualitative analysis were performed. Before the analysis, all patient contours were visually verified by two medical physicists to identify whether any systematic or random differences existed and to check the consistency and quality of the data. The main detected systematic differences were in contouring protocols used clinically within our department and those used for the model development. For example, kidneys were contoured with hilus by all auto-segmentation software tools and without manually. All the organs with variable shapes and sizes (e.g. bladder, bowel) sometimes failed completely (e.g., misplacing the OAR etc.). Software C didn’t include a bowel contouring model. The clinic-specific findings from the visual evaluation are summarized in Supplementary Data II.

In the second phase, a medical physicist calculated median values of the clinicians’ ratings. An interdisciplinary group discuss the results and reached the ultimate decision of the most suitable software, based on the quantitative and qualitative results, user experience and the tools' ability to delineate additional structures that are currently not delineated clinically.

In the third phase, the operational implementation of the selected software was prepared by interdisciplinary and site-specific focus meetings attended by physicians working in relevant tumour group, one RTT, and the involved medical physicists. The meetings consisted of a review of the evaluation and a case-by-case discussion. A guideline catalogue with descriptive instructions for each structure was developed and training of the involved personnel was prepared.

### 3.2 Quantitative and qualitative analysis

All auto-segmented contours were imported into the RayStation (v11A, RaySearch Laboratories AB, Stockholm, Sweden) treatment planning system. Every contour set of each patient was imported in a separate case.

Two quantitative geometrical parameters were calculated using built-in scripts in RayStation: Dice Similarity Coefficient (DSC) describing the volumetric similarity between two structures, ranging from 0 to 1 where 1 indicates perfect overlap between contours; Hausdorff Distance (HD) quantifying the distance between two contours where the lower HD the better agreement [3], [18]. Additionally, average dose (D_mean_) and maximum dose (D1%) for the original and recalculated plan on the new contours from each auto-segmentation model were reported for analysis. Lastly, a qualitative evaluation using a physician's blind rating of three auto-segmented contour sets and manual contours was performed [19]. The rating was done by ten different radiation oncologists and residents, with each patient case rated by at least two different physicians (see Table 1). None of them had prior knowledge of the evaluated case's segmentation source (software or human). Ratings of 4 and 3 corresponded to acceptable contours with no or minor modifications, respectively, and rating of 2 and 1 to major changes or rejections, respectively.

### 3.3 Inter-observer variability

To quantify the inter-observer variability [1], [12], three medical doctors delineated OARs for ten female pelvis, ten male pelvis, and two other doctors delineated 15 abdomen cases. The clinical manual contour was used as a reference for the inter-observer study.

### 3.4 Statistical analysis

For each OAR, the hypothesis that the contours generated by the auto-segmentation models were at least equivalent in qualitative metrics to the manual reference contours was tested. For quantitative metrics, we tested the hypothesis to determine whether significant differences existed among the results from the various software tools. The geometric and dose/volume metrics comparison results were tested with Kruskal Wallis, followed by posthoc Dunn with Bonferroni correction, to identify the best-performing model.

We assessed the correlation (Spearman's rank correlation) between quantitative metrics and MD ratings to determine their predictive value for clinical acceptance.

## 4 Results

### 4.1 Quantitative evaluation: Geometric and dose/volume metrics results

**Fig. 2:**
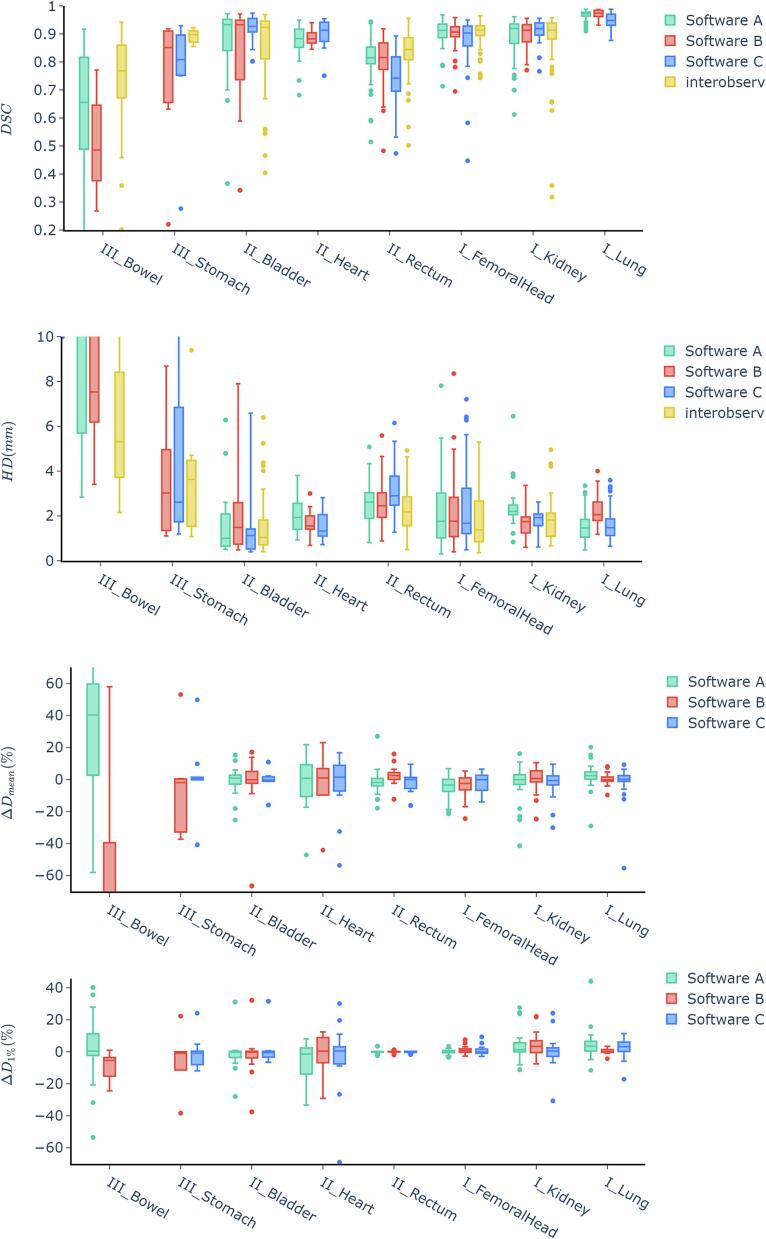
Results of the *DSC*, *HD* (in mm), *D*_mean_ and *D*_1%_ comparison between software tools A, B and C for the thorax, abdomen, and pelvis region. *D*_mean_ and *D*_1%_ are given as relative differences (%) compared to the original plan. The roman numerals indicate the classification according to chapter 2.5 in categories of class I, class II and class III.

**Fig. 3:**
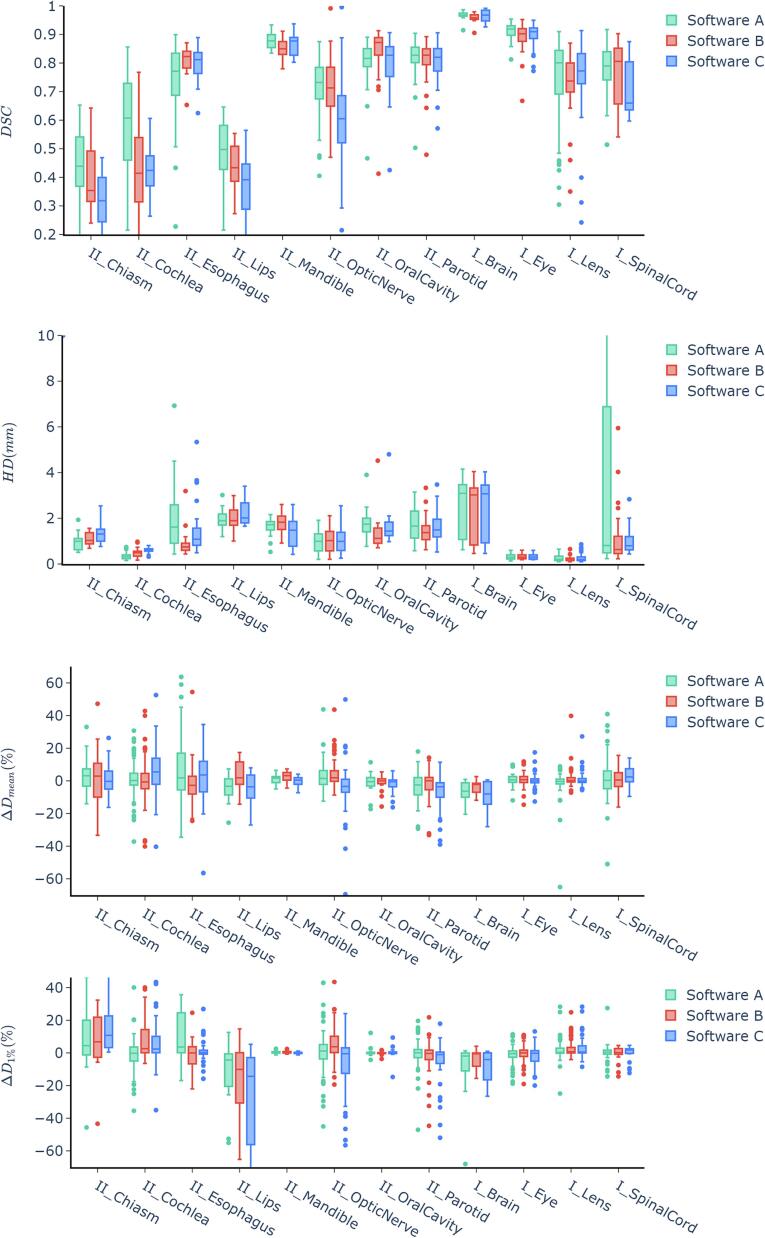
Results of the the *DSC*, *HD* (in mm), *D*_mean_ and *D*_1%_ comparison between software tools A, B and C for the central nervous system, head and neck, and upper digestive tract. *D*_mean_ and *D*_1%_ are given as relative differences (%) compared to the original plan. The roman numerals indicate the classification according to chapter 2.5 in categories of class I, class II and class III.

The combined average Dice Similarity Coefficient (DSC) scores for all organs at risk (OARs) were 0.80 ± 0.10 for Software A, 0.75 ± 0.10 for Software B, and 0.74 ± 0.11 for Software C. Some OARs had DSC scores below the acceptable 0.7 level in each software; these included the chiasm, pituitary, and cochlea for Software A, the lacrimal gland and bowel for Software B, and the optic nerve for Software C. Conversely, specific OARs exceeded an average DSC of 0.9, notably the brain and lung in all three software programs. For a more detailed analysis, see Fig. 2 and Fig. 3.

In Hausdorff Distances (HD) were the lowest in lens, eye, pituitary, and cochlea, while the highest in the bowel and liver. Software B and C did not contour the spinal cord in abdomen cases, contributing to higher HD values for Software A in that region due to comparison with incomplete manual contour.

Dose/volume metrics showed average dose differences within ± 5 % for most OARs with exceptions for heart, bowel, liver, and brain in Software A, bowel, heart, bladder and lacrimal gland in software B and brain, lacrimal gland, heart, optic nerve, cochlea and brainstem in software C.

Statistically significant differences in the geometric and dosimetric results of software A, B, and C are reported in detail in Supplementary Data III. No significant differences were found for half of the investigated OARs (10/20). Only in the rectum did the DSC scores between the different software tools differ. For the rest, typically, two software tools performed similarly, while one was significantly worse, except in the cochlea, where software A outperformed the others.

The geometric evaluation of the interobserver study on a subset of pelvis and abdomen patients showed a similar variance in the geometric scores of inter-observer contours compared to auto-contours.

### 4.2 Qualitative evaluation

The results of the physician ratings are shown in Table 2, a graphical representation of the ratings in a radar plot can be found in Supplementary Data IV. Only for bowel, manual contours were rated significantly higher than result of any auto-segmentation tool. Detailed statistical analysis of the results is in Supplementary Data III.

**Table 2:** Data of the qualitative evaluation showing median MD ratings and the quartiles (0.25–0.75).

OAR	manual		Software A		Software B		Software C	
	Median	Q1-Q3	Median	Q1-Q3	Median	Q1-Q3	Median	Q1-Q3
*Class I*								
Brain	4	4–4	4	4–4	3	3–3	4	4–4
Eye	4	4–4	4	4–4	4	4–4	4	4–4
FemoralHead	3	3–4	4	4–4	3	3–4	3	3–3
Kidney	4	3–4	4	3–4	3	2–4	4	3–4
Lens	4	4–4	4	4–4	4	4–4	4	4–4
Liver	4	3–4	4	3–4	3	3–4	3	3–3
Lung	4	3–4	4	3–4	4	4–4	3	3–3
SpinalCord	4	4–4	3	3–4	4	4–4	4	3–4
Thyroid	4	4–4	4	3–4	4	4–4	3	2–3
*Class II*								
Rectum	3	3–4	3	3–4	2	2–3	2	2–3
Lips	3	3–4	4	4–4	3	3–4	3	3–4
Mandible	4	3–4	4	4–4	4	4–4	4	4–4
Heart	3	3–4	3	3–4	3	3–4	3	3–4
Bladder	3	2–3	3	2–4	3	1–3	3	3–4
Esophagus	4	4–4	3	2–4	4	4–4	3	3–4
OpticNerve	4	3–4	3	3–3	3	3–4	4	4–4
Chiasm	4	3–4	4	3–4	3	3–3	4	4–4
Brainstem	4	4–4	4	4–4	4	3–4	4	3–4
Cochlea	4	4–4	4	4–4	4	3–4	4	3–4
Parotid	4	3–4	4	4–4	4	4–4	4	4–4
OralCavity	4	3–4	3	3–3	3	3–4	3	3–3
*Class III*								
Stomach	3	3–4	–	–	3	1–3	2	1–3
Bowel	3	2–3	2	2–2	2	1–2	–	–

The kidneys, femoral heads, eyes, and brainstem of software A showed the highest median rating. The spinal cord and the heart of software B was rated better than any other contours. Software C achieved the highest overall rating for the optic nerve, chiasm and brain.

In the heart (p = 0.26), bladder (p = 0.05), brainstem (p = 0.08), and lens (p = 0.28), the results of the ratings did not significantly differ among any software or the manual contours.

### 4.3 Dependency of metrics

Table 3 shows cross-calibration matrices. Spearman coefficients indicated little to no correlation for most classes but showed moderate to strong correlations for class III OARs.

**Table 3:** Correlation table showing the Spearman correlation coefficients of the three software tools with respect to the OAR classification (I, II or III) and the different quantitative metrics (DICE, HD and dose) vs. MD rating.

Software	Class	Spearman correlation coefficient		
		DICE	HD	Dose/volume
A	I	0.16	0.12	0.05
	II	0.19	0.31	0.05
	III	0.36	0.21	0.31
	combined	0.55	0.40	0.17
B	I	0.01	0.12	0.11
	II	0.12	0.49	0.04
	III	0.53	0.48	0.65
	combined	0.41	0.46	0.19
C	I	0.11	0.57	0.06
	II	0.15	0.47	0.16
	III	0.85	0.44	0.44
	combined	0.03	0.55	0.05

## 5 Discussion

We have developed a comprehensive method for evaluation und understanding the performance of auto-segmentation tools based on qualitative, quantitative and dosimetric parameters, and multi-disciplinary discussion. The qualitative rating by MD was considered the most relevant. The most important lesson learned was that none of the metrics can be blindly used for the decision of the contour's acceptability. Ideally, all three methods should be employed. Visual evaluation is necessary to understand the differences to the clinical practice. The quantitative assessment provides baseline parameters for tracing the quality of the contours after updates. The results of the analysis were used for treatment-site independent clinical workflow definition with minimal manual corrections. Our overall positive conclusion regarding the clinical implementation of deep learning-based auto-segmentation is in line with recently published literature. Our study covers all clinically relevant sites, compared to more narrow studies for head-and-neck [6], [10], [12], breast [13], prostate [12], [14], thorax [15], [16], central nervous system [12], or cervix [17].

The method was used for a comparison of auto-segmented OARs using three different software tools. In most cases, all three software tools produced good, and sometimes even superior results compared to manual segmentations. Their outcome was mostly similar. Software A performed better in the abdomen and pelvis, while software C's performance was rated higher for the brain region, with software B falling somewhere in the middle. However, in some organs at risk (OARs), the results were systematically inferior and did not meet the clinical requirements of radiation oncologists. This agrees with previous studies [6], [10], [12], [15], [16].

Along with AI-based models for the automatic generation of treatment plans [20], [21], [22] and strategies for workflow optimization [23], auto-segmentation plays a vital role in automizing the treatment planning process and making it more efficient. A recent survey found, that while the perceived impact of auto-contouring was positive, only a minority was using it on a larger scale [24]. Arguments have been made that extensive upfront validation and testing are needed prior to the clinical implementation of AI-based auto-segmentation tools [25]. Therefore, when introducing commercial auto-segmentation software into clinical practice, we propose a multidisciplinary evaluation to understand the performance and limitations of the employed software.

The decision on implementing such a software tool is not straight-forward and strongly depends on the focus of the clinic and treatment sites to be auto-segmented. Moreover, we believe that it is important to understand the evaluation parameters and their relevancy for the decision and clinical impact. We have demonstrated that the geometric and dose/volume results did not necessarily correspond with good clinical ratings by radiation oncologists. While higher DSC, HD, and D_mean_ scores typically yielded better physician ratings, there were several exceptions where good performance in these measures did not translate to high ratings. On the other hand, organs with a low DSC (and high HD) sometimes resulted very positive MD ratings (e.g., chiasm). The DSC showed a strong correlation with physician rating only for class III organs and might be useful to identify structures not useful for clinical implementation. As pointed out by others [3], the DSC is dependent on the organ size. Although the quantitative analysis did in general not correlate with the physician rating, it is still useful to acquire these data in the implementation phase to have a baseline quantitative parameter for QA purposes, e.g., after updates.

A comparison between the dose/volume metrics of the manual and auto-segmented contours can provide a useful information on the relevancy of the observed contour differences. However, the location of the tumor significantly influences the sensitivity of dose differences, a factor that certainly must be accounted for when reporting such findings [26].

The site-specific focus group meetings led to categorizing each OAR into three groups, forming the foundation for new delineation protocols detailed in Supplementary Data V, with all RTTs trained on these classifications.

Class I represents OARs where the models perform very well. These contours can be directly accepted after a plausibility review. If during this review minimal corrections would be identified, these require no action. Class II OARs often require small adjustments due to differences in the auto-segmentation models and the anatomical definitions used in the clinic. Lastly, class III OARs are typically clinically unacceptable and may require more time and effort to modify than manually delineate the contours from scratch. However, a visual inspection should be performed before contour approval, even for class I contours. Atypical cases, e.g., implants, anatomical anomalies (e.g., nephrectomy) or large tumors close to the contoured OAR, may cause any model to fail [11].

An important aspect of implementing auto-segmentation in a department is the level of corrections necessary for auto-segmented structures to be deemed clinically acceptable. In the site-specific discussions with multidisciplinary teams, the auto-segmented contour was often accepted without correction, even with systematic differences from manual contours. For example, kidneys were contoured without renal hilus clinically but with hilus in all software. Esophagus and heart had different craniocaudal extension definitions. While the difference in dose/volume parameters was relatively small, the impact was discussed in the multidisciplinary meetings to understand whether the different OAR definitions can be clinically accepted. We want to stress that any adaptations in the institutional contouring protocol must be carefully reviewed, as these changes will impact the planning process, from planning objectives to clinical goals and constraints, and reporting in e.g. clinical studies.

Another important outcome of this analysis was the standardization of the contouring within the department. The multidisciplinary discussion revealed differences in OAR definition across different treatment site groups (e.g., spinal cord and spinal canal). Here, the more consistent structures from the software tools will improve intra-departmental homogeneity. While not the subject of our study, our observations were in line with other studies that investigated interobserver variability among AI models [12].

When integrating auto-segmentation tools into clinical practices, a multitude of factors, beyond the segmentation outcomes, play a pivotal role in determining the choice of software. These include costs, user-friendliness, automation efficiency, seamless integration with existing systems, ease of maintenance, additional feature sets, and the roadmap for future capabilities. It is impossible to provide universal guidelines or even a weighted prioritization rank list, but we recommend incorporating these clinic-specific considerations into your decision-making process to ensure optimal utilization.

We acknowledge several study limitations. First, due to resource constraints, additional manual contours were created only for a subset of patients, preventing variation correction like averaging multiple expert segmentations. Second, we didn't perform a time comparison between manual and auto-segmentation, which requires more resources. Third, the quantitative parameters DSC and HD employed in this study have shortcomings such as the volume dependence of the DSC and the HD not being robust against outliers. Nevertheless, they were easily accessible using the TPS and were therefore used. Recently, other metrics such as surface DSC and HD95% have been proposed to compensate these issues. [27], [28]. Fourth, dose re-optimization on auto-contours was not conducted, which might impact dose/volume results when planning is based on AI-derived contours. Finally, we assessed only two key dose/volume parameters (D_mean_ and D_max_), which may not fully represent all treatment sites. We note that commercial software is continually improving, including forthcoming MR-based models that may enhance OAR accuracy.

In summary, emphasizing the significance of clinical acceptance as a crucial factor in evaluating AI segmentation models, it is strongly recommended to establish a thorough workflow for the interdisciplinary assessment of auto-segmentation software prior to its implementation in clinical settings. Such a workflow will provide valuable insights into the suitability of the chosen software and the necessary adaptations required for institutional protocols. Additionally, such a workflow helps establishing guidelines for the use of the software and the determination of a baseline for regular QA of the software.

## Declaration of Competing Interest

The authors declare that they have no known competing financial interests or personal relationships that could have appeared to influence the work reported in this paper.
