# Supplementary material for: Clinical Implementation and Evaluation of Auto-Segmentation Tools for Multi-Site Contouring in Radiotherapy
Source: Phys Imaging Radiat Oncol. 2023 Nov 17;28:100515. doi: 10.1016/j.phro.2023.100515 (PMC10726238; doi:10.1016/j.phro.2023.100515)
Supplement: Supplementary Data 1 [file mmc1.docx]

Supplementary Data

Supplementary Data I: Overview of commercial software analyzed in this study

| Software | Alias | Integration | Type |
| --- | --- | --- | --- |
| Annotate (v1.10.0, Therapanacea, France) | Software A | Non-intrusive | Full body |
| Limbus Contour (v1.5.0, Limbus AI, Canada) | Software B | Non-intrusive | Site specific |
| RayStation (v 11B, RaySearch Laboratories, Sweden) | Software C | Manually triggered | Site specific |

Supplementary Data II: This is an example of notes after the visual inspection in preparation of the site-specific interdisciplinary meetings. Note that these discoveries are very clinic-specific and might vary, depending on the guidelines for manual OAR delineations.

| Site | Finding |
| --- | --- |
| Brain | The cranio-caudal borders of the brainstem differed in the three tools. Software C did provide a connection between the optic nerve and the chiasm, whereas the other tools did not. |
| Head&neck | The definition of the larynx was different in the three tools. |
| Thorax | The esophagus was not included in the breast template in software B and was therefore missing in 8 out of 15 thorax patients The cranial extent of the esophagus was slightly inconsistent and differed from the clinical contours. |
| Abdomen | The delineation of OARs differed the most among the software tools in this region. Mainly due to tumours negatively impacting the size and shape of OARs. Stomach is not contoured with software tool A. The bowel had the worse results. It was not contoured by Software C. The software tools A and B delineated the bowel bag and, in both cases, with lot of uncertainty. Software B, in addition, delineated the bowel loops. |
| Female pelvis | Software C did not have a model for female pelvis. EMBRACE patients had very good results. Other tumour sites were more difficult. The most problematic was segmentation of bowel, same as in abdomen region. Kidneys were contoured with hilus compared to clinical protocol by all software tools. Bladder often differed in cranial or caudal direction by all software. |
| Male pelvis | Software C failed to delineate the rectum when a rectal balloon was present. All three software worked the best for prostate cancer but had worse results for other indications (e.g. bladder). The most problematic was segmentation of bowel, same as in abdomen region. |

Supplementary Data III: Statistical analysis of the MD ratings (left) and DSC (right). Posthoc Dunn's test was performed when significant differences across the manual and software generated MD ratings were found. Yellow coloring indicates significant differences between the software tools and/or manual contours.

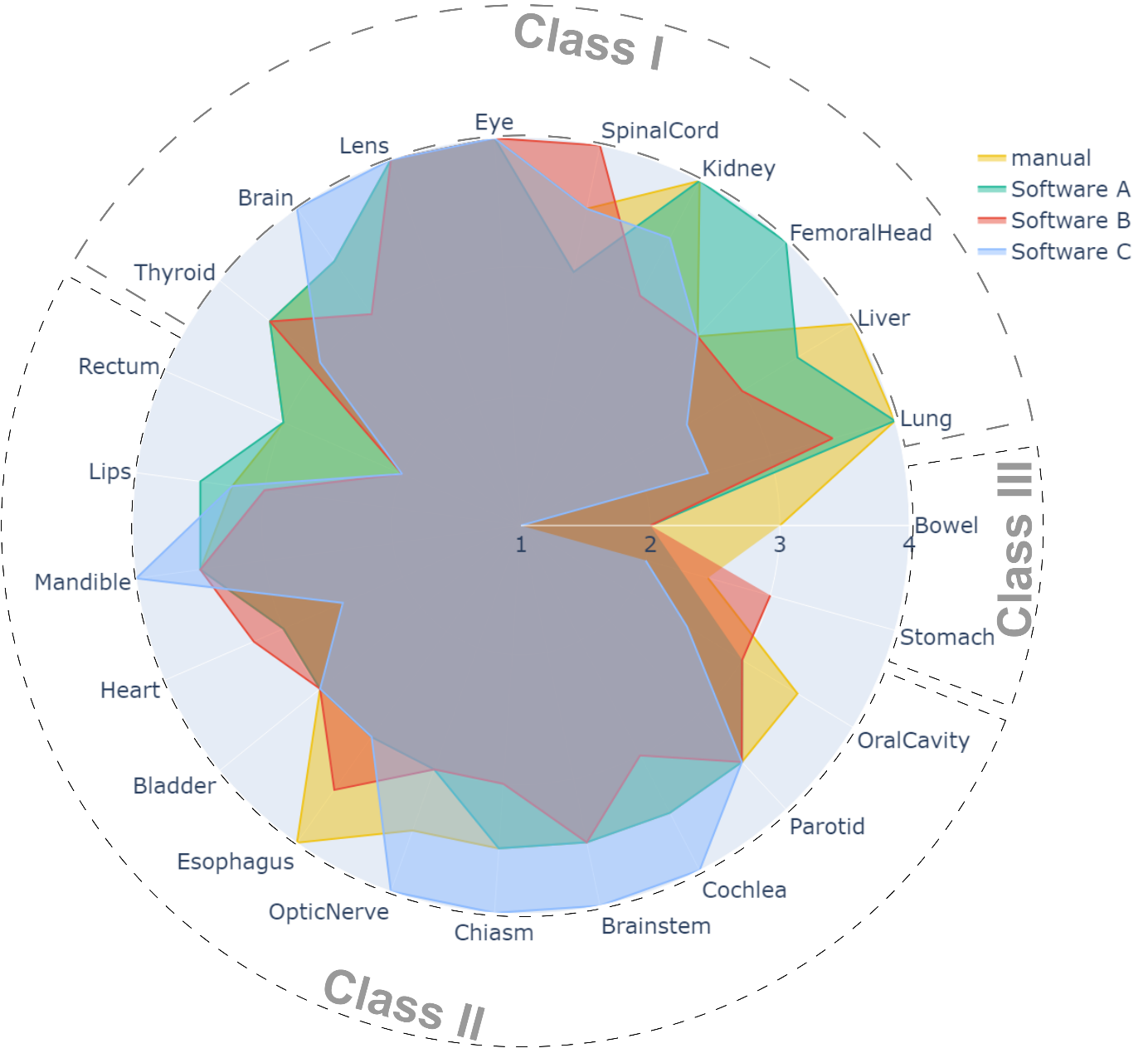


Supplementary Data IV: Mean physician ratings for OARs. The roman numerals indicate the classification according to chapter 3.5 in categories of class I, class II, and class III. Note that software C did not produce contours for the bowel.

Supplementary Data V: An overview of the clinical guidelines on autosegmentation. Based on the interdisciplinary discussion, three institutional-specific classes were defined for the candidate software tool. Class I contours can be directly used without modification after a brief visual inspection; they typically exhibit minor in-slice segmentation deviations. Class II contours necessitate adjustments, due to issues such as compromised anterior-posterior lengths or significant shape underrepresentation near the treated volume. Class III contours are unsuitable for immediate clinical use and require manual recontouring due to partial or complete misplacement or large errors across all slices, making the correction process more time-consuming than manual segmentation.

| OAR | Class | Advice |
| --- | --- | --- |
| Anal Canal | II | Control in detail and correct. Merge together with rectum using ROI algebra and create Anorectum (same as clinically used rectum contour so far). |
| Bladder | II | Control in detail and correct. |
| Body | III | Do not use autosegmentation |
| Bowel | III | Do not use autosegmentation |
| Brain | I | Check plausibility. Do not improve minor deviations. |
| Brainstem | II | Control in detail and correct. |
| Chiasm | II | Control in detail and correct. Important! Chiasm and optic nerve must be connected. |
| Cochlea | II | Control in detail and correct. |
| Esophagus | II | Control in detail and correct. Important! The caudal end must be connected to stomach. |
| Eye | I | Check plausibility. Do not improve minor deviations. |
| Femoral Head | I | Check plausibility. Do not improve minor deviations. |
| Heart | II | Control in detail and correct. Important! Keep the cranial length of the heart. Do not shorten it. |
| Humerus | I | Check plausibility. Do not improve minor deviations. |
| Kidney | I | Check plausibility. Do not improve minor deviations. Important! Keep hilus in the contour. |
| Larynx | I | Check plausibility. Do not improve minor deviations. Important! Correct cranial length. |
| Lens | I | Check plausibility. Do not improve minor deviations. |
| Liver | I | Check plausibility. Do not improve minor deviations. |
| Lips | II | Control in detail and correct. Important! Control and improve left and right side. |
| Mandible | II | Control in detail and correct. Important! Do not correct teeth. |
| Lung | I | Check plausibility. Do not improve minor deviations. |
| Optic Nerve | II | Control in detail and correct. Important! Chiasm and optic nerve must be connected. |
| Oral Cavity | II | Control in detail and correct. Important! The muscles of the floor of mouth can stay in the contour. Base of tongue must be corrected. |
| Parotid | II | Control in detail and correct. |
| Rectum | II | Control in detail and correct. Merge together with rectum using ROI algebra and create Anorectum (same as clinically used rectum contour so far) |
| Sigmoid | III | Do not use autosegmentation |
| Submandibular gland | I | Check plausibility. Do not improve minor deviations. |
| Spinal Canal | I | Check plausibility. Do not improve minor deviations. |
| Spinal Cord | I | Check plausibility. Do not improve minor deviations. |
| Thyroid | I | Check plausibility. Do not improve minor deviations. |
| TM Joint | I | Check plausibility. Do not improve minor deviations. |
